# *Gcm2* regulates the maintenance of parathyroid cells in adult mice

**DOI:** 10.1371/journal.pone.0210662

**Published:** 2019-01-24

**Authors:** Taku Yamada, Norifumi Tatsumi, Akane Anraku, Hideaki Suzuki, Sahoko Kamejima, Taketo Uchiyama, Ichiro Ohkido, Takashi Yokoo, Masataka Okabe

**Affiliations:** 1 Division of Nephrology and Hypertension, Department of Internal Medicine, The Jikei University School of Medicine, Tokyo, Japan; 2 Department of Anatomy, The Jikei University School of Medicine, Tokyo, Japan; Nanjing Medical University, CHINA

## Abstract

Glial cells missing homolog 2 (GCM2), a zinc finger-type transcription factor, is essential for the development of parathyroid glands. It is considered to be a master regulator because the glands do not form when *Gcm2* is deficient. Remarkably, *Gcm2* expression is maintained throughout the fetal stage and after birth. Considering the *Gcm2* function in embryonic stages, it is predicted that *Gcm2* maintains parathyroid cell differentiation and survival in adults. However, there is a lack of research regarding the function of *Gcm2* in adulthood. Therefore, we analyzed *Gcm2* function in adult tamoxifen-inducible *Gcm2* conditional knockout mice. One month after tamoxifen injection, *Gcm2-*knockout mice showed no significant difference in serum calcium, phosphate, and PTH levels and in the expressions of calcium-sensing receptor (*Casr*) and parathyroid hormone (*Pth*), whereas Ki-67 positive cells were decreased and terminal deoxynucleotidyl transferase (TdT) dUTP Nick-End Labeling (TUNEL) positive cell number did not change, as compared with those of controls. Seven months after tamoxifen injection, *Gcm2-*knockout mice showed shrinkage of the parathyroid glands and fewer parathyroid cells. A significant decrease was noted in *Casr-* and *Pth*-expressing cells and serum PTH and Ca levels, whereas serum phosphate levels increased, as compared with those of controls. All our results concluded that a reduction of *Gcm2* expression leads to a reduction of parathyroid cell proliferation, an increase in cell death, and an attenuation of parathyroid function. Therefore, we indicate that *Gcm2* plays a prominent role in adult parathyroid cell proliferation and maintenance.

## Introduction

Calcium (Ca) ions are indispensable for neurotransmission, muscle contraction, blood coagulation, and bone formation. A failure of serum Ca homeostasis thus causes death. For this reason, serum Ca ion concentrations are strictly maintained, principally by the parathyroid glands [1]. In humans, these glands comprise parathyroid hormone (PTH)-producing chief cells and oxyphilic cells. Calcium-sensing receptors (CASRs) on the surfaces of chief cells are capable of sensing a decrease in Ca ions, prompting the parathyroid glands to produce and secrete PTH [2]. In contrast, elevated Ca detected by CASRs and/or vitamin D receptor (VDR) stimulated by vitamin D each or both suppressed PTH production [3–5]. PTH releases Ca that is stored in bone as calcium phosphate into the blood, whereas released Ca is reabsorbed in the renal tubule. Two processes maintain the Ca concentration [6]. In addition, PTH promotes and stabilizes the excretion of phosphorus (P) by suppressing reabsorption of P in the renal tubules that is released from the bone with Ca.

As a zinc finger-type transcription factor, glial cells missing homolog 2 (GCM2) is known to be a master regulator for embryonic development of parathyroid glands. Developmentally, *Gcm2* is first expressed in the third pharyngeal pouch at E9.5 and subsequently in the parathyroid region of developed parathyroid/thymus primordium at E11.5 in mice [7]. At E13, the parathyroid–thymus primordium divides itself into the parathyroid glands and the thymus.

In *Gcm2*-null mice, at E12 to E12.5, parathyroid precursor cells die of apoptosis [8] and have no parathyroid glands and die soon after birth [9]. As a result, since the parathyroid glands are not formed in *Gcm2*-null mice, GCM2 is believed to be essential for development of the parathyroid glands and survival at the earliest stage after organ specification during the embryogenesis [9–11]. In addition, GCM2 is predicted to be a key regulator of parathyroid function in mineral homeostasis. A series of analyses indicate that *Gcm2* subsequently regulates serum calcium concentration by regulating *Casr* expression [12–14] and promoting PTH secretion along with *MafB* and *GATA3*.

*Gcm2* is expressed throughout the fetal stages and after birth specifically in parathyroid cells. However, it is unclear whether GCM2 functions in adult parathyroid cells.

Considering reports on *Gcm2* [7–15], we hypothesized it maintains parathyroid cell survival and differentiation throughout adulthood. But no studies have examined the function of *Gcm2* in adult parathyroid cells, since *Gcm2*-knockout mice do not have parathyroid glands.

In this study, we analyzed *Gcm2* function in the adult parathyroid glands using *Gcm2* conditional knockout mice with tamoxifen-inducible Cre–LoxP system and investigated whether *Gcm2* has an important function in the adult parathyroid glands.

## Materials and methods

### Equipment, animals, and generation of the *Gcm2*-floxed allele

A Microscope Imager D1 (ZEISS) and Camera AxioCam MRc5 (ZEISS) was used to obtain images of parathyroid gland sections that have been processed with H.E staining, *in situ* hybridization, Proliferation Cell Nuclear Antigen (PCNA) immunostaining and Ki-67 immunostaining. LSM 800 Airyscan was used for the terminal deoxynucleotidyl transferase (TdT) dUTP Nick-End Labeling (TUNEL) assay.

**Fig 1:**
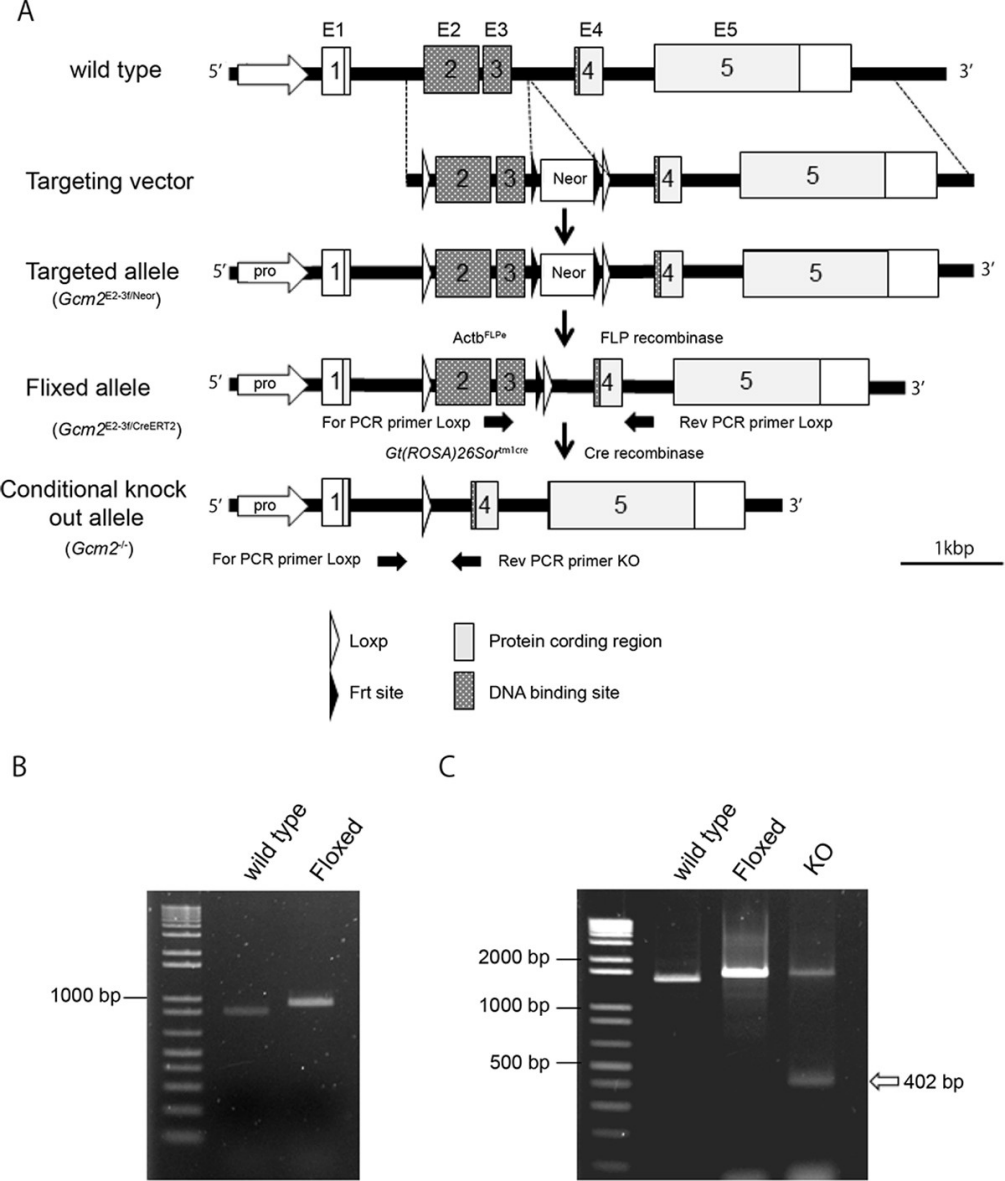
Construction of the *Gcm2* conditional knockout mouse model. (A) *Gcm2* has five exons: exon 2 and 3 contain the DNA binding site (black box). At the targeting vector construction, a *loxP* sequence was inserted in intron 1 (306 bp upstream of exon 2), and a *loxP* and *FRT*-flanked neo-cassette sequence was inserted in intron 3 (333 bp downstream of exon 3). The schema indicates the wild-type, targeting vector, targeted allele, floxed allele, and conditional knockout allele from the top to the bottom. The positions of the PCR primers are shown by the black thick arrows. (B) Genomic PCR in wild-type (C57BL/6N 846 bp) and floxed (*Gcm2*^*E2-3flNeor/ E2-3flNeor*^ 922 bp) mice. (C) Genomic PCR in the wild-type (1454 bp), floxed (1522 bp), and *Gcm2* knockout (1522 bp and 402 bp) alleles. The short band of the KO schema indicates the KO band (white arrow).

This study was approved by the Institutional Animal Care and Use Committee of the Jikei University School of Medicine. All animals were maintained and treated in accordance with the guidelines and accepted standards of humane animal care. Mice were sacrificed with 120 mg/kg of pentobarbital sodium by peritoneal injection. Blood sampling and sample collections were performed after the mice had been euthanized; the mice experienced no pain. The *Gcm2*^*+*^*/*^*E2-3fl-Neor*^ mice were generated and provided by RIKEN BRC through the National Bio-Resource Project of MEXT, Japan. The BAC clone for the targeted region RP24-307019 was obtained from Open Biosystems. Briefly, the *loxP* and *FRT*-flanked neomycin resistance gene cassette (*FRT-PGK-gb2-neo-FRT*) (Gene Bridges) were inserted using a BAC Subcloning Kit by Red/ET Recombination (Gene Bridges). Exons 2 and 3 of *Gcm2* were flanked by *loxP* sites (Fig 1A). The first *loxP* was inserted into intron 1 (306 base pairs [bp] upstream of exon 2), before the *FRT*-flanked neo-cassette and the second *loxP* were inserted into intron 3 (333 bp downstream of exon 3). The targeting vector was constructed by sub-cloning the *loxP* region flanked by exons 2 and 3 of the *Gcm2* gene with homologous arms into the DT-A-pA vector (RIKEN BRC).

The resultant mouse chimeras were mated with C57BL/6N female and F1 hybrid *Gcm2*^*E2-3fl*/-^ mice were genotyped by genomic polymerase chain reaction (PCR) to contain a *loxP* site in cloned DNA. The primers used were: *Gcm2* exon 3–4 forward 5′-GGATACCCTGTCACCAACTTCTG-3′ and *Gcm2* exon 3–4 reverse 5′- GTCTCTTGAGGGCACTTCTTCTG-3′. Homozygous mice were developed by brother and sister mating over twenty generations. Mutant mice were mated with FLP mice (*Actb-FLPe ∙ C57BL/6N*) to delete Neo^r^. The *Gcm2* ^*E2-3fl/E2-3fl*^ mice were mated with *Gt(ROSA)26Sor*^*tm1crERT2e/tm1creERT2*^ mice (Jackson Laboratory stock #3368809) to produce *Gcm2* ^*E2-3fl/E2-3fl*^*;Gt(ROSA)26Sor*^*+/tm1CreERT2*^. Finally, *Gcm2*^*loxP/loxP*^*;Gt(ROSA)26Sor*^+*/tm1CreERT2*^were mated with *Gcm2*^*E2-3fl/E2-3fl*^;*Gt(ROSA)26Sor*^+*/tm1CreERT2*^ and *Gcm2*^*E2-3fl/E2-3fl*^;*Gt(ROSA)26Sor* ^*tm1CreERT2/tm1CreERT2*^ were produced.

### Knockout mouse assay

Tamoxifen solution (10 mg/ml) was prepared by dissolving tamoxifen in ethanol and mixing it with sunflower oil at a ratio of 1:4. To produce adult conditional knockout mice, we injected 40 mg/kg tamoxifen into 8-week-old *Gcm2*^*E2-3fl/E2-3fl*^;*Gt(ROSA)26Sor* ^*tm1CreERT2/tm1CreERT2*^ mice intraperitoneally for five days. As the control, *Gcm2*^*E2-3fl/E2-3fl*^;*Gt(ROSA)26Sor* ^*tm1CreERT2/tm1CreERT2*^ mice received solution injection without tamoxifen were used. To produce knockout embryonic mice, *Gcm2*^*E2-3fl/E2-3fl*^*;Gt(ROSA)26Sor* ^*tm1CreERT2/tm1CreERT2*^ pregnant mice were injected with 1.0 mg of tamoxifen at E6.5 and E7.5. and the embryos were collected at E12.5 (referred as KOE12.5). As the control, *Gcm2*^*E2-3fl/E2-3fl*^*;Gt(ROSA)26Sor* ^*tm1CreERT2/tm1CreERT2*^ E12.5 mice witch harvested form pregnant mouse received solution injection without tamoxifen were used.

### Genotyping

Genotypes were determined by genomic DNA PCR, using the following primer sets by KOD FX (TAKARA) (Fig 1B). The PCR probes to screen LoxP alleles were forward primer 5′-GGATACCCTGTCACCAACTTCTG-3′ and reverse primer 5′-GTCTCTTGAGGGCACTTCTTCTG-3 ′, with forward primer annealing of exon 3 and forward primer annealing of exon 4 (wild-type 846 bp, floxed 922 bp). The PCR probes to knock out alleles were forward primer 5 ′-AGTGGGAAAGCATTCTGACCA-3′ and reverse primer 5 ′-AGGGTCACAGTATCTATGAGGCT-3′ (wild-type, 1454 bp; floxed, 1522 bp; and knockout, 402 bp) (Fig 1C).

### Biochemical analyses

Whole blood was collected by cardiac puncture and was centrifuged to collect serum. Total serum Ca, P, were measured using SPOTCHEM D-02 (ARKRAY). PTH was measured using a Mouse PTH 1–84 ELISA Kit (Immutopics). Blood samples were separately collected each week for up to four weeks and each month for up to seven months after tamoxifen administration. We measured serum Ca, P, and PTH levels one month after tamoxifen administration (1MP.I) and seven months after tamoxifen administration (7MP.I) in each blood sample (including control samples).

### Histological analysis

For the preparation of *Gcm2* knockout mice embryonic day (E) 12.5 (KOE12.5), 8-week-old *Gcm2*^*E2-3fl/E2-3fl*^*;Gt(ROSA)26Sor* ^*tm1CreERT2/tm1CreERT2*^ mice were mated, and pregnant mice were injected with 1.0 mg of tamoxifen at E6.5 and E7.5. The embryos were collected at E12.5, fixed in 4% paraformaldehyde, and embedded in paraffin, before 5 μm sections were prepared.

*Gcm2* knockout in 8-week-old adult mice was performed by continuous peritoneal injection of 40 mg/kg of tamoxifen over five days. We euthanized the mice at one month or seven months post tamoxifen injection (1MP.I and 7MP.I, respectively). Controls were euthanized at the same ages as knockout mice. The tracheas, thyroids, and parathyroids were dissected en bloc from mice, fixed in 4% paraformaldehyde, and embedded in paraffin before preparing 6 μm sections. Slides were stained with hematoxylin and eosin for histological analysis of the parathyroid gland.

### Section *in situ* hybridization

Paraffin section *in situ* hybridization was performed at KOE12.5, 1MP.I, and 7MP.I as previously described [16,17]. The 6-μm paraffin sections of the parathyroid and 5-μm paraffin sections of embryos were hybridized with digoxigenin-labeled RNA probes at 0.5 μg/ml. The *Gcm2* probes (forward 5′-TTTGACCACTTCCGGGAGTG-3′ and reverse 5′- ACCCTGTCACCAACTTCTGG-3′) corresponded with exons 2 and 3 of *Gcm2*. Probes of *Pth*, *Casr*, and *Foxn1* were cloned using the following primers, respectively: forward 5′-TCAGTTTGTGCATCCCCGAA-3′ and reverse 5′-CTCTTCCTCACGGGTTTCCC-3′ for *Pth*; forward 5′-GAAGCAACAGCAACCACTGG-3′ and reverse 5′-GTCATTGCTCTTCTGGGCCT-3′ for *Casr*; and forward 5′-CACTACCTGTCTCCTATGCCAC-3′ and reverse 5′-GATGCTTAAGACAGTTGACCGC-3′ for *Foxn1*.

### Immunohistochemistry

The immunohistochemical expression of Ki-67 and PCNA was studied in paraffin sections of the parathyroid tissues from 1MP.I and 7MP.I mice using 6 μm paraffin sections with the avidin–biotin–peroxidase complex method (VECTOR LABORATORIES, VECTATION ABC Kit, biotin 1/200). Rabbit anti-Ki-67 monoclonal antibody (Thermo Fisher Scientific, Ki-67 Antibody Monoclonal SP6, 1/1000) and rabbit anti-PCNA polyclonal antibody(abcam, Anti-PCNA antibody ab18197, 1/2000) was used for each Ki-67 staining and PCNA staining, and the nucleus was stained with hematoxylin. Quantification of parathyroid cell proliferation (% of cell proliferation) was measured by counting each the total number of Ki67-positive nuclei and PCNA-positive nuclei in the section of the parathyroid tissues. This number was then divided by the total number of parathyroid cells.

### Terminal deoxynucleotidyl transferase (TdT) dUTP nick-end labeling assay

Apoptotic signals were detected by TUNEL assay. The TUNEL assay was performed with 6 μm paraffin-embedded parathyroid tissue sections, as previously described [18]. Nucleus staining was performed with DAPI (4′,6-diamidino-2-phenylindole). Quantification of parathyroid cell death (i.e., the TUNEL-positive ratio) was obtained by counting the total number of TUNEL-positive nuclei in the whole sections of the parathyroid tissues and dividing them by the total number of the parathyroid cells in those sections.

### Statistical analyses

All results are expressed as mean ± SD. All results were analyzed using Mann–Whitney U-test. Differences were considered statistically significant at P < 0.05.

## Results

### Preparation of tamoxifen-induced *Gcm2* conditional knockout mice

The *Gcm2* gene has five exons, but only exons 2 and 3 contain the DNA binding site [9,19,20]. We induced *Gcm2*^*E2-3*fl-Neo^ with exons 2 and 3 flanked by *loxP* sites (i.e., floxed) to knockout the *Gcm2* DNA binding site through the Cre–LoxP system (Fig 1A). Genomic PCR was performed to analyze flox-site integration and demonstrated wild-type (C57BL/6N 846 bp) and floxed (*Gcm2*^*E2-3fl-Neor/ E2-3fl-Neor*^ 922 bp) bands, confirming that the flox site was successfully inserted (Fig 1B). The neomycin resistant gene cassette was deleted by mating with FLP mice. We crossed the floxed mouse (*Gcm2* ^*E2-3fl/E2-3fl*^) to mouse Gt(ROSA)26Sor ^tm1CreERT2/tm1CreERT2^ Tyj and prepared *Gcm2*^E2-3fl/ E2-3fl^;Gt(ROSA)26Sor ^tm1CreERT2/tm1CreERT2^ mice (Fig 1A).

When mice were eight weeks old, we injected 40 mg/kg tamoxifen intraperitoneally for five days to delete exons 2 and 3, before performing genomic PCR to analyze gene deletion at the flox site. Consequently, the knockout DNA was confirmed at 402 bp, the floxed was at 1522 bp, and the wild-type was at 1454 bp, indicating that the Cre recombination has been correctly performed (Fig 1C). Knockout efficiency of *Gcm2* was 80%–90%.

**Fig 2:**
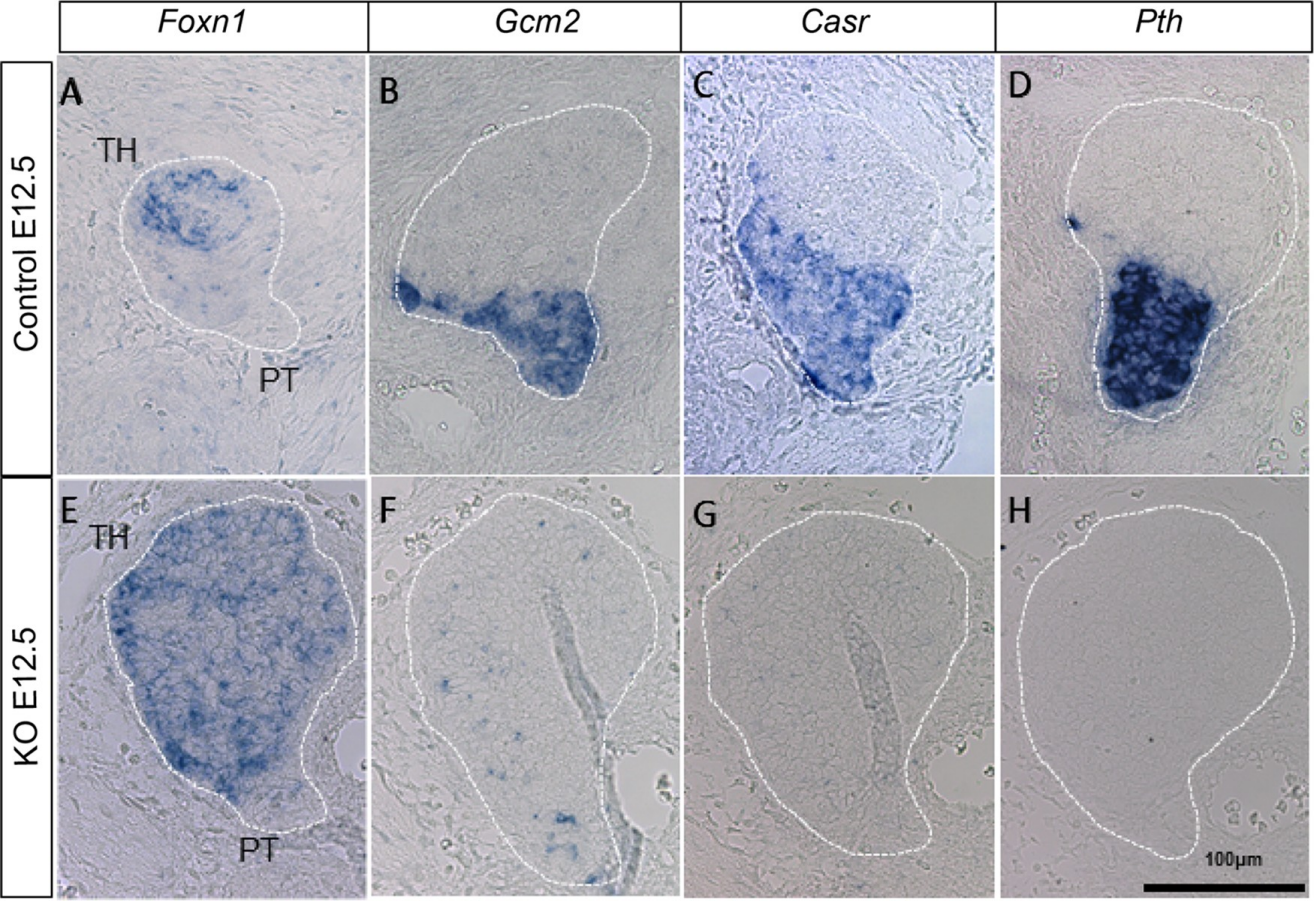
Expression patterns for marker genes in *Gcm2* conditional knockout mice. *In situ* hybridization of parathyroid markers *Gcm2* (B, F), *Casr* (C, G), and *Pth* (D, H) and thymus marker genes *Foxn1* (A, E) in the primordial tissue (thymus and parathyroid) of E12.5 mice in control (A–D) and *Gcm2* conditional knockout (E–H) groups. The dotted lines indicate the thymus–parathyroid primordium. The thymus (TH) and parathyroid (PT) regions are shown in A and E, respectively. Scale bars indicate 100 μm.

We also needed to confirm whether *Gcm2* was functionally knocked out in *Gcm2*^E2-3fl/ E2-3fl^;Gt(ROSA)26Sor ^tm1CreERT2/tm1CreERT2^ mice. The parathyroid destined domain is distinguished at E10.5 in the thymus–parathyroid primordium as a *Gcm2-*expressing domain, with the primordium ultimately separating into a parathyroid gland and thymus lobe from E12.5 to E13.5. [21–23]. Considering that *Casr* and *Pth* are not found in the parathyroid gland of the *Gcm2*-knockout mouse[8,18], a similar phenotype should be seen in in *Gcm2*^*E2-3fl/E2-3fl*^*;Gt(ROSA)26Sor* ^*tm1CreERT2/tm1CreERT2*^ mice. Therefore, tamoxifen was intraperitoneally injected into E 6.5 pregnant mice intraperitoneally for 2 days, and the expression of *Gcm2*, *Casr*, *Pth* and thymic marker *forkhead box N1* (*Foxn1*) was analyzed at E 12.5. We found that *Foxn1* expressions were identified the thymus–parathyroid primordium in both the KOE12.5 and control mice (Fig 2A and 2E). *Gcm2*, *Casr*, and *Pth* were expressed in the thymus–parathyroid gland primordium of control mice but not in that of knockout mice (Fig 2B, 2D, 2F and 2H). These results showed that *Gcm2*^*E2-3fl/ E2-3fl*^*;Gt(ROSA)26Sor* ^*tm1CreERT2/tm1CreERT2*^ mice injected with tamoxifen displayed the same phenotype to that of *Gcm2*-knockout mice [8], indicating that our conditional knockout of *Gcm2* was successful.

### Functional analysis of *Gcm2* in adult parathyroid gland

**Fig 3:**
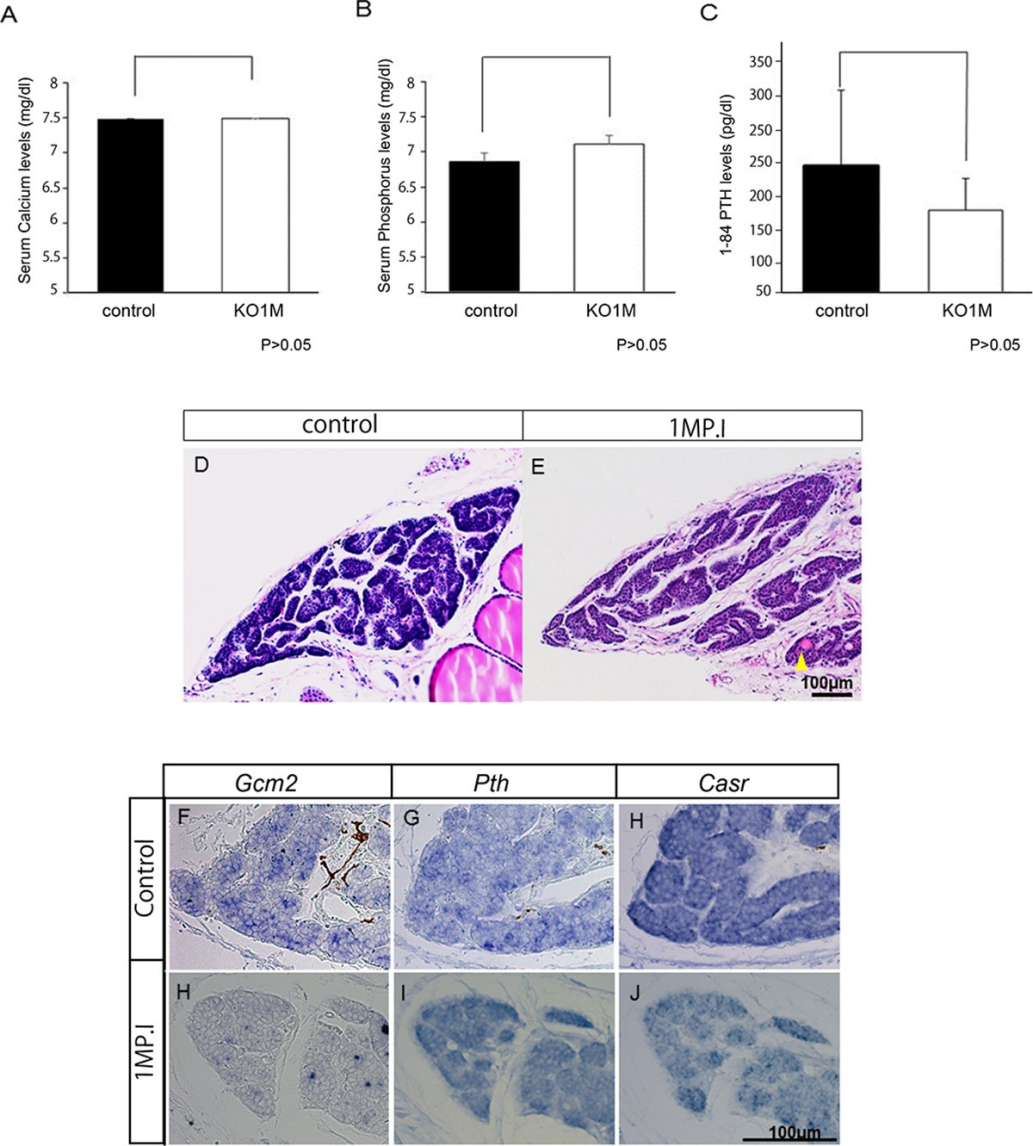
Biochemical and histological analyses of 1MP.I mice. (A–C) The graphs indicate the biochemical results for serum Ca, P, and 1–84 PTH concentrations for control mice (white bar, n = 15) and 1MP.I mice (black bar, n = 14) (U-test, P > 0.05). (D, E) Histology of the parathyroid gland of control mice (D) and 1MP.I mice (E) stained with hematoxylin and eosin. Yellow arrowhead (E) indicates acinar structures surrounded by cells in the parathyroid glands. (F–K) *In situ* hybridization of *Gcm2* (F and I), *Pth* (G and J), and *Casr* (*H* and *K*) in the parathyroid gland of control mice (F–H) and 1MP.I mice (I–K). All scale bars were 100 μm.

After confirming that conditional *Gcm2* knockout was possible, we repeated the procedure in adult *Gcm2*^*E2-3fl/ E2-3fl*^*;Gt(ROSA)26Sor* ^*tm1CreERT2/tm1CreERT2*^ mice. We injected tamoxifen intraperitoneally into 8-week-old adult *Gcm2*^*E2-3fl/ E2-3fl*^*;Gt(ROSA)26Sor* ^*tm1CreERT2/tm1CreERT2*^ mice to achieve *Gcm2* knock out. The serum Ca, and P levels are known to change in *Gcm2* knockout mice [9]. Therefore, we measured serum Ca and P levels to analyze the influence of the lack of *Gcm2* on parathyroid function in mineral homeostasis, we collected blood samples each week for up to four weeks after tamoxifen administration (WP.I) (S1A Fig). *Gcm2* conditional KO mice had levels of serum Ca and P similar to those in controls, in samples from all weeks (S1C Fig). We then considered that PTH secretion changes would probably precede serum Ca and P concentration changes and measured the serum PTH concentrations one month after tamoxifen administration (1MP.I mice). We found the serum PTH tended to be lower in those mice than in controls, but the difference was not statistically significant (Fig 3A–3C).

The parathyroid glands of 1MP.I mice were then excised. Since the parathyroid gland is very small and buried in the thyroid gland, change is difficult to observe, so we observed parathyroid morphologies with sections. When the gross parathyroid tissues were compared in control and knockout mice, there were no obvious differences in size (S2C Fig), though many knockout mice had some follicular structures surrounded by cells in the parathyroid glands that were not present in controls (Fig 3D and 3E, N = 14). Subsequently, we analyzed the expressions of *Casr*, *Pth*, and *Gcm2* by *in situ* hybridization of tissues from the parathyroid glands of 1MP.I mice to gain a better understanding. In control mice, each gene was expressed throughout the parathyroid tissues; however, although there were few *Gcm2*-expressing cells remaining in the parathyroid tissues of 1MP.I mice, the expressions of *Pth* and *Casr* were almost entirely comparable to those in the control mice (Fig 3F–3K).

**Fig 4:**
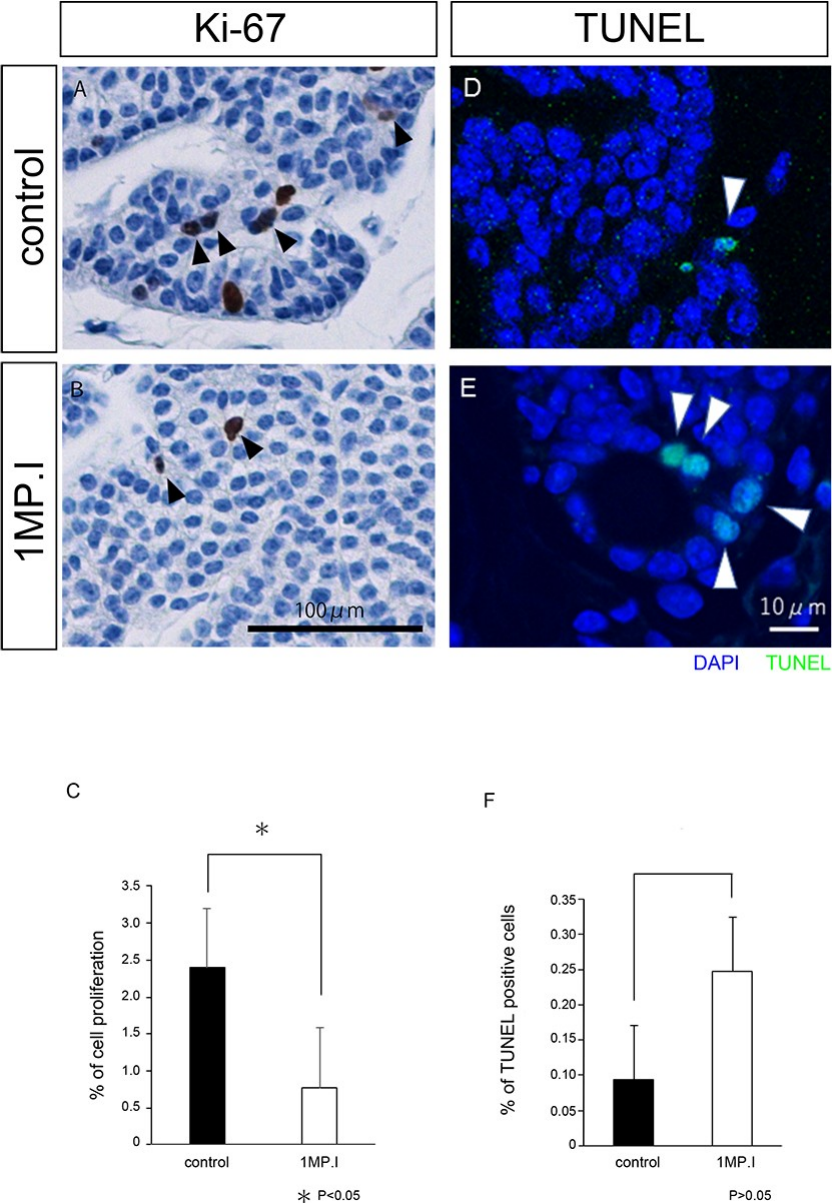
Analysis of cell proliferation and death in 1MP.I mice by Ki-67 and TUNEL immunostaining. (A, B) Ki-67 immunostaining of the parathyroid gland tissues in control mice (A) and 1MP.I mice (B). Brown-colored cells are Ki-67-positive cells, and black arrowheads indicate Ki-67-positive parathyroid cells (Scale bar = 100 μm). (C) Control and 1MP.I tissues had Ki-67-positive cell ratios of 2.4% (n = 9) and 0.77% (n = 7), respectively (U-test, *P < 0.05). (D, E) TUNEL staining of the parathyroid gland tissues in control mice (D) and 1MP.I mice (E). TUNEL-positive cells are indicated in green and all cells were stained blue with DAPI. The white arrowheads indicate the TUNEL-positive parathyroid cells (scale bar = 10 μm). (F) Control and 1MP.I tissues had TUNEL-positive cell ratios of 0.1% (black bar, n = 6) and 0.25% (white bar, n = 5), respectively (U-test, *P > 0.05).

We also checked cell proliferation and cell death in the parathyroid tissues of *Gcm2*-knockout mice. Immunostaining was carried out with Ki-67 and PCNA to analyze cell proliferation. Parathyroid cells positive for Ki-67 were found in 2%–2.5% of all the parathyroid cells in controls, but in less than 1% of the knockout mice parathyroid cells (black arrowhead) (Fig 4A–4C). In addition, parathyroid cells positive for PCNA were found in 0.7–1.0% of all the parathyroid cells in controls, but in 0.2% of the knockout mice parathyroid cells (black arrowhead) (S2A and S2C Fig). Both analyses indicated thus that there were decreased numbers of proliferating parathyroid cells. The number of cell deaths in the control and knockout mice was compared using TUNEL assay (Fig 4D–4F). In the control mice, TUNEL-positive cells accounted for approximately 0.1% of all the parathyroid cells, and there was no unevenness in their distribution (Fig 4F). By contrast, in 1MP.I mice, TUNEL-positive cells accounted for approximately 0.25% of all the parathyroid cells, and these were mainly present around the acinar structures (Fig 4E and 4F). Cell death was increased in the parathyroid glands of *Gcm2-*knockout mice as compared with that in control mice, but there was no significant difference. Most dead cells in 1MP.I mice were found only around the acinar structures (Fig 4D and 4E).

In the 1MP.I mice, there was no significant change in parathyroid gland area/cell number ratio (pixel/number) or gene expression patterns, but by this stage, acinar structures have formed, cell death have increased, and cell proliferation have decreased.

### Long-term functional analysis of *Gcm2* knockout in adult mice

**Fig 5:**
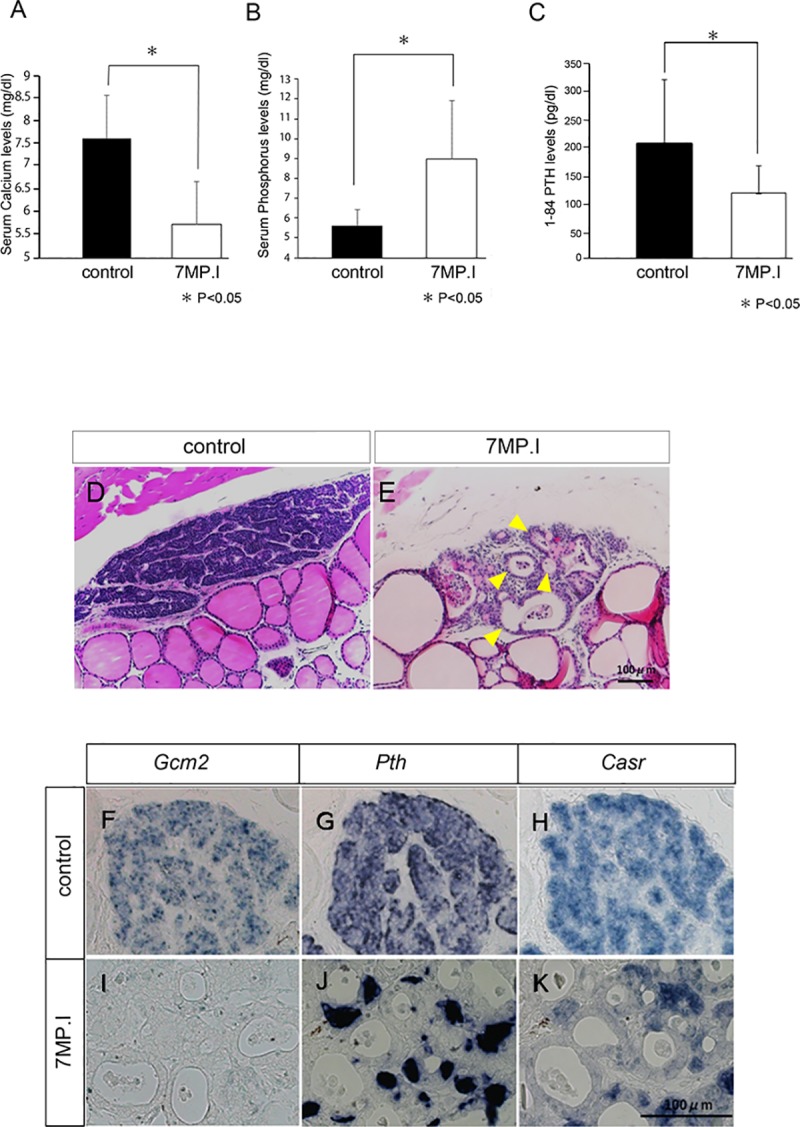
Biochemical and histological analyses in the 7MP.I mice. (A–C) Graphs show the serum levels of Ca (A), P (B), and 1–84 PTH (C) in control (black bar, n = 7) and 7MP.I mice (white bar, n = 6) mice (U-test, *P < 0.05). (D, E) Parathyroid gland sections stained with hematoxylin and eosin for control (D) and 7MP.I (E) mice. Multiple acinar structures can be seen in the parathyroid glands of 7MP.I mice (yellow arrowhead). (F–K) *In situ* hybridization for parathyroid marker genes: *Gcm2* (F, I), *Pth* (G, J), and *Casr* (H, K) in control (F–H) and in 7MP.I mice (I–K) (scale bars = 100 μm).

We investigated the effect of decreasing cell proliferation in knockout mice over a longer time by injecting 8-week-old *Gcm2*^*E2-3fl/E2-3fl*^*;Gt(ROSA)26Sor* ^*tm1CreERT2/tm1CreERT2*^ mice with tamoxifen and measuring serum Ca and P each month up to seven months after tamoxifen administration (S1B Fig). We found that serum Ca, and P levels did not differ in control mice seven months after tamoxifen administration (S1B Fig). However, the serum Ca levels were significantly decreased and the serum P levels were significantly increased in *Gcm2-*knockout mice seven months after tamoxifen administration (7MP.I mice) compared with those in control mice (Fig 5A and 5B). To explore the causes for the serum Ca and P level changes, we measured PTH concentrations in 7MP.I mice. There was a significant decline in PTH concentrations in 7MP.I mice (Fig 5C). We predicted changes in gene expression and parathyroid morphology changes from serum Ca and PTH reduction, serum P increase in 7MP.I mice. Therefore, we further investigated morphological changes and gene expression changes in 7MP.I mice parathyroid glands analyzed by those sections.

First, we performed hematoxylin and eosin staining to analyze it detail morphology (Fig 5D and 5E). With the control of 7MP.I, samples retained normal form with dense cellular organization (Fig 5D). By contrast, the 7MP.I samples were markedly abnormal: the normal structure of the parathyroid glands was barely present, the number of cells was significantly reduced, and the total tissue size was reduced (Fig 5E and S3B Fig). In addition, the lumens of acinar structure were expanded and the number of acinar structures increased, and only a few parathyroid cells were present in the tissue surrounding acinar structures (Fig 5E, yellow arrowhead).

Second, we analyzed the gene expressions of *Casr*, *Pth*, and *Gcm2* in the parathyroid gland of 7MP.I mice by *in situ* hybridization (Fig 5F–5K). In the control mice, these genes were expressed in the parathyroid tissues (Fig 5F–5H), whereas in 7MP.I mice, *Gcm2* expression was not observed and the expressions of *Casr* and *Pth* were markedly reduced (Fig 5I–5K).

**Fig 6:**
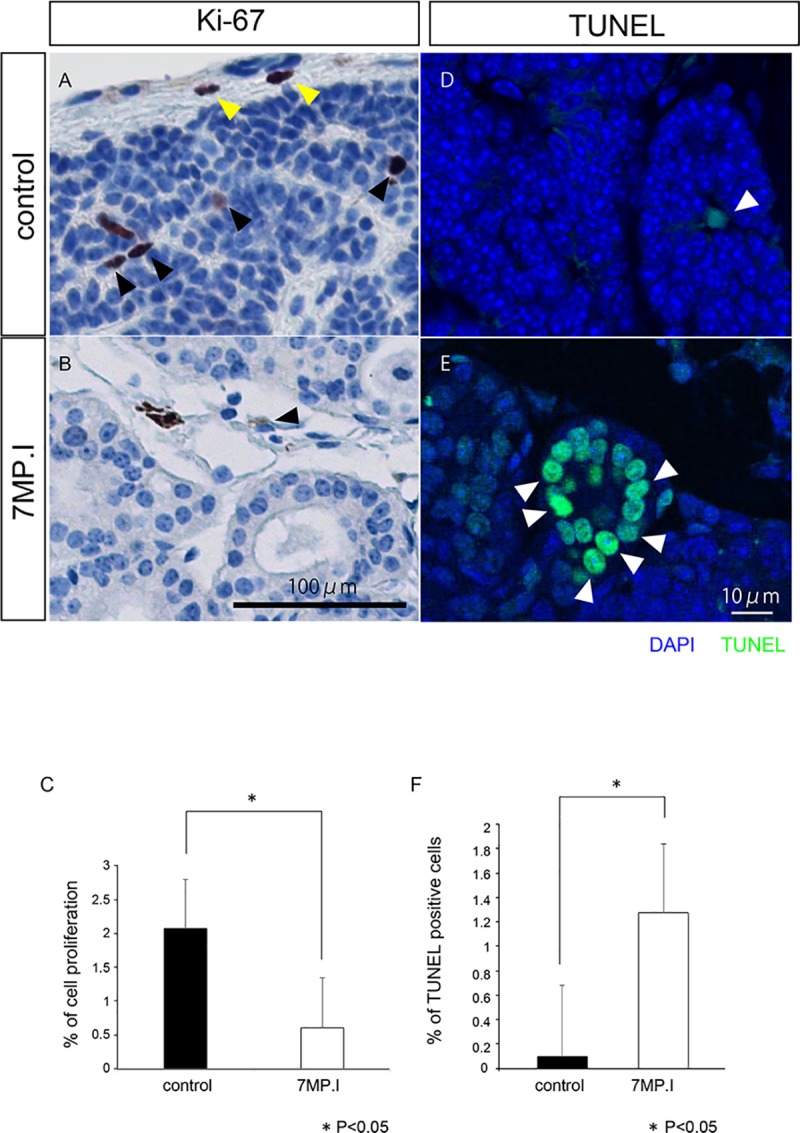
Analysis of cell proliferation by Ki-67 immunostaining in 7MP.I cells. (A, B) Ki-67 immunostaining in the parathyroid glands of control (A) and 7MP.I (B) mice (black arrowheads indicate Ki-67-positive parathyroid cells, and yellow arrowheads indicate Ki-67-positive stromal cells; scale bar = 100 μm). (C) Control and 7MP.I tissues had Ki-67-positive cell ratios of 2.1% (black bar, n = 6) and 0.6% (white bar, n = 5), respectively (U-test, *P < 0.05) (S2D–S2F Fig for PCNA). (D, E) TUNEL staining of the parathyroid gland tissues in control mice (D) and 7MP.I mice (E). TUNEL-positive cells are indicated in green and all cells were stained blue with DAPI. The white arrowheads indicate the TUNEL-positive parathyroid cells (scale bar = 10 μm). (F) Control and 7MP.I tissues had TUNEL-positive cell ratios of 0.09% (black bar, n = 6) and 1.27% (white bar, n = 5), respectively (U-test, *P > 0.05) (See also S4 Fig).

Third, we examined the proliferation of parathyroid cells by Ki-67 and PCNA in 7MP.I mice. In control tissues, Ki-67-positive cells were observed in both parathyroid cells (black arrowhead) and stromal cells (yellow arrowhead) (Fig 6A), whereas in 7MP.I mice tissues, Ki-67 positive cells were observed (black arrowhead) (Fig 6B). As was the case for 1MP.I mice, Ki-67-positive cells remained diminished in the 7MP.I mice as compared with those in control mice (Figs 4A, 4C, 6A and 4C). We found PCNA-positive parathyroid cells in 1.0% of all the parathyroid cells in controls, but only in 0.2% of the same cells in 7MP.I mice (black arrowhead) (S2D–S2F Fig). These results indicate that the *Gcm2-*knockout mice had decreased numbers of proliferating parathyroid cells in both the Ki-67 and the PCNA analyses.

Finally, we performed a TUNEL assay using the *Gcm2-*knockout mice to analyze cell-death levels. TUNEL-positive cell ratios were increased in 7MP.I mice (1.27%) compared with those in the control mice (0.09%) (Fig 6D–6F). These results indicate that the *Gcm2-*knockout mice had more parathyroid cell deaths in the TUNEL analysis than the control mice. Thus, we concluded that *Gcm2* knockout in adult mice resulted in a loss of normal parathyroid function and of cell proliferation for parathyroid maintenance.

## Discussion

The function of *Gcm2* is indispensable for the proliferation and differentiation of parathyroid gland cells during fetal development [9,24], and *Gcm2* is expressed in the parathyroid cells [9–11]. However, whether *Gcm2* continues to be expressed and is involved in cell proliferation in the adult parathyroid glands is uncertain. Therefore, we performed a functional analysis of *Gcm2* in adult mice using a conditional knockout model.

In our study, we established a new model for *Gcm2* function in adult mice and demonstrated that a reduction of *Gcm2* caused a reduction of parathyroid cell proliferation, with reduced parathyroid gland size, and degradation of the physiological activity of the adult parathyroid glands. To date, three lines of *Gcm2*-knockout mice have been prepared. Two of these mice have knockouts that span exons 2 to 5 and lack *Gcm2* [9,24] and they, therefore, did not form the parathyroid glands. Consequently, they were unsuitable to investigate parathyroid gland function in adults. In the other strain of *Gcm2*-knockout mice, the Cre–loxP system knocks out *Gcm2* by mating Cre mice postnatally; however, this only affects exon 2 [20]. We constructed a model to knock out exons 2 and 3 where the DNA binding site of *Gcm2* was present. Therefore, unlike the previously reported models, our mice were characterized by having a complete defective *Gcm2* function, making them ideal for analyzing effects of *Gcm2* knockout in adults.

Regarding *Casr* expression, it was thought in previous reports that a reduction of *Gcm2* decreased *Casr* expression. It has been reported that *Gcm2* binds to the DNA binding site of *Casr* where it directly controls *Casr* expression and *Casr* expression was decreased by *Gcm2* deficiency in developmental stage mice [8]. Our result showed a similar result in the *Gcm2* knockout embryo mice. Cell experiments have also shown that *Casr* expression was decreased by *Gcm2* knockdown [13]. However, our *in vivo* result showed that *Casr* was expressed even though *Gcm2* was lacking at 1MP.I. This result thus suggests that matured parathyroid cell could express *Casr* without *Gcm2* in adult mice. Other factors other than Gcm2 may control the expression of *Casr* in parathyroid cells. For example, in humans, a DNA binding site for vitamin D is present in the promoter region of *CASR* and the site is also known to be preserved in rodents [25,26]. In turn, several studies have shown that vitamin D activates *Casr* expression in the parathyroid glands [26]. Since vitamin D was included in the feed for the mice in our experiments, VDR may have regulated *Casr* expression independently from *Gcm2*. Experiments with vitamin D deficient diets will reveal a more precise mechanism for *Casr* expression.

Regarding *Pth* expression, it is known that *GATA3*, *MafB*, and *Gcm2* work cooperatively and that *Pth* can be expressed in cultured cell experiments even when one of these is deficient [14,19,27]. In our experiments, *Pth* was expressed in *Gcm2*-knockout mice even one month after tamoxifen administration. Therefore, *Pth* expression was probably maintained by factors other than *Gcm2*. Our biochemical analyses showed decreased PTH levels in the *Gcm2*-knockout mice after one month, but the Ca and P levels remained unaffected. However, after seven months, the expression of *Pth* in parathyroid tissues, the serum PTH level, and the serum Ca all decreased, and the serum P increased, as expected in cases of clinical hypoparathyroidism. Human parathyroid glands contain chief (active and inactive) and oxyphilic cells, but only chief cells produce *Pth*. In addition, inactive chief cells are thought to differentiate into oxyphilic cells [2]. Thus, the cells lacking *Pth* expression in the parathyroid gland of *Gcm2*-knockout mice may be oxyphilic. If so, Gcm2 may be involved in the maintenance of chief cells in mature parathyroid glands.

The results on *Casr* and *Pth* indicate that both their expressions remained after *Gcm2* became deficient, but other mechanisms may help maintain their expressions levels independently of the presence of *Gcm2*. *Gcm2* may also be involved in the maintenance of parathyroid cells, in particular of their gene expression. Further elucidation of the mechanisms for regulating *Casr* and *Pth* will be key to understanding the functional maintenance of the parathyroid glands.

The deficiency of *Gcm2* during the developmental phase causes cell death of parathyroid progenitor cells and thus leads to absence of the gland. The absence of normal differentiation of parathyroid cells was thought to be devoid of any response to survival signals in the environment [8]. After *Gcm2* knockout in the mature parathyroid gland we found no difference in the numbers of TUNEL-staining positive cells as compared with those in the control mice 1MP.I, but the number of TUNEL-staining positive cells was significantly increased in the 7MP.I. However, the parathyroid gland was always present in the mice during the investigated period. This is because cell death did not occur in the entire gland and because even if *Gcm2* was deficient the apoptosis rate increased very slowly. Our results indicate that *Gcm2* is not a factor for cell survival in matured parathyroid cells (unlike its role during development). In addition, we observed the proliferation of cells at one and seven months after tamoxifen injection. As a result, after *Gcm2*-knockout the parathyroid showed lower proliferation ability of the cells as compared to that in control cells, but the reduction of cell proliferation rates after one and seven months in the *Gcm2*-knockout were similar. It is likely that the decrease in cell proliferation due to *Gcm2* deficiency may not occur rapidly but occur at a constant rate and that there may be very few but constant cell proliferation and cell death even in normal parathyroid glands. Consequently, our results do not rule out a role for *Gcm2* in regulation of normal parathyroid cell proliferation and cell death. This may explain the elevated expression of *Gcm2* in secondary hyperparathyroidism [15]. In addition, it is known that abnormalities of *Gcm2* occur in primary hypoparathyroidism, hyperparathyroidism, and parathyroid tumors with parathyroid cell proliferation [28,29]. Therefore, *Gcm2* regulates the slow proliferation of the parathyroid cells.

Factors other than *Gcm2* related to cell proliferation are known. Morito et al (2017) reported that cell proliferation decreased in secondary hyperparathyroidism due to drug-induced renal failure in *MafB*-knockout mice and that *MafB* may play a role in the hyperplastic cell proliferation of secondary hyperparathyroidism [6]. They also reported that adult *MafB*-knockout mice without secondary hyperparathyroidism maintained their normal parathyroid function, tissue morphology, and cell proliferation, suggesting that *MafB* was not involved in maintaining normal proliferation [6]. Future research will be needed to elucidate how *Gcm2* and *MafB* are related to proliferation of mature parathyroid glands.

For the first time, our present study showed that a reduction of *Gcm2* caused a reduction of cell proliferation and changed the pattern of cell death in adult parathyroid glands, the event that caused a decline in the parathyroid tissues. Therefore, *Gcm2* affects cell proliferation and maintenance in adult parathyroid glands. It is our hope that our present study will open a door to our further understanding of *Gcm2* functions in cell cycles of normal parathyroid cells.

## Supporting information

S1 Fig (A, B) Experimental time course of blood sampling after tamoxifen injection. (C) Weekly serum Ca and P concentrations for up to four weeks after administration of tamoxifen. Serum Ca and P levels were all similar to those in controls at all weeks. (D) Monthly serum Ca and P concentrations for up to seven months after administration of tamoxifen (7MP.I). Serum Ca levels were significantly decrease and serum P levels were significantly increase in 7MP.I mice compared with those in control mice.
(TIF)

S2 Fig (A, B) PCNA immunostaining of the parathyroid gland tissues in control mice (A) and in one months after administration of tamoxifen (1MP.I) mice (B). Brown-colored cells are PCNA-positive cells, and black arrowheads indicate PCNA-positive parathyroid cells (Scale bar = 100 μm). (C) Control and 1MP.I mice tissues had PCNA-positive cell ratios of 1.02% (n = 5) and 0.21% (n = 5) (U-test, *P < 0.05). (C, D) PCNA immunostaining in the parathyroid glands of control (C) and 7MP.I mice (D). Black arrowheads indicate PCNA-positive parathyroid cells (scale bar = 100 μm). (E) Control and 7MP.I mice tissues had PCNA-positive cell ratios of 1.01% (black bar, n = 5) and 0.23% (white bar, n = 5), respectively (U-test, *P < 0.05).
(TIF)

S3 Fig (A) Control and 1MP.I had parathyroid gland area/cell number ratios at 904 (n = 6) and 954 (n = 6), respectively (U-test, *P < 0.05). (B) The area/cell number ratios of parathyroid glands of control and 7MP.I had 929 (n = 7) and 556 (n = 4), respectively (U-test, *P < 0.05).
(TIF)

S4 Fig Box-plot diagram of TUNEL-positive cells percentages. Results were varied, but we found no statistically significant differences between control and 1MP.I mice.
(TIF)
