# Supplementary figures and images for: Gcm2 regulates the maintenance of parathyroid cells in adult mice
Source: PLoS One. 2019 Jan 24;14(1):e0210662. doi: 10.1371/journal.pone.0210662 (PMC6345461; doi:10.1371/journal.pone.0210662)

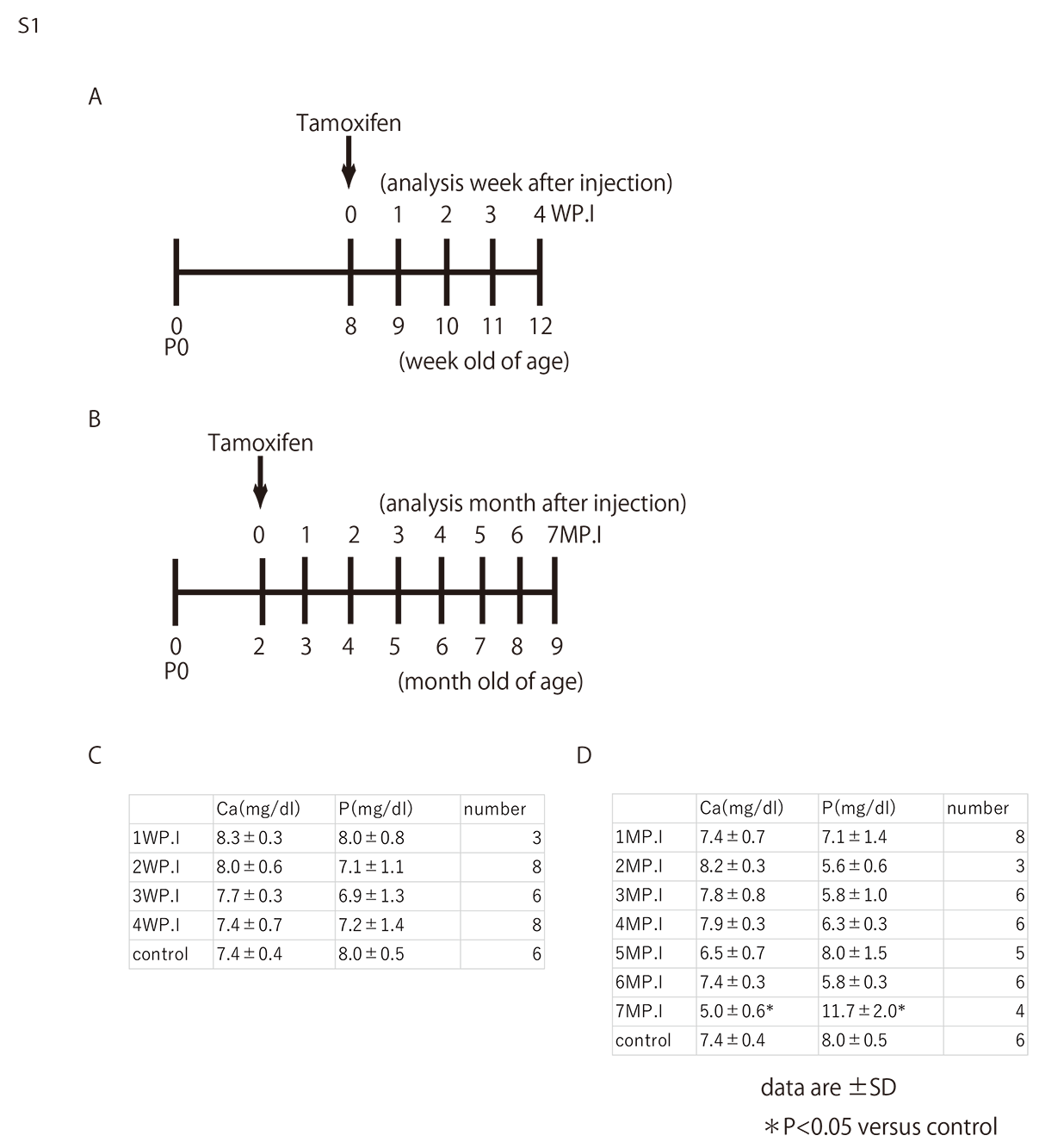

Supplement: S1 Fig — (A, B) Experimental time course of blood sampling after tamoxifen injection. (C) Weekly serum Ca and P concentrations for up to four weeks after administration of tamoxifen. Serum Ca and P levels were all similar to those in controls at all weeks. (D) Monthly serum Ca and P concentrations for up to seven months after administration of tamoxifen (7MP.I). Serum Ca levels were significantly decrease and serum P levels were significantly increase in 7MP.I mice compared with those in control mice. (TIF) [file pone.0210662.s001.tif]

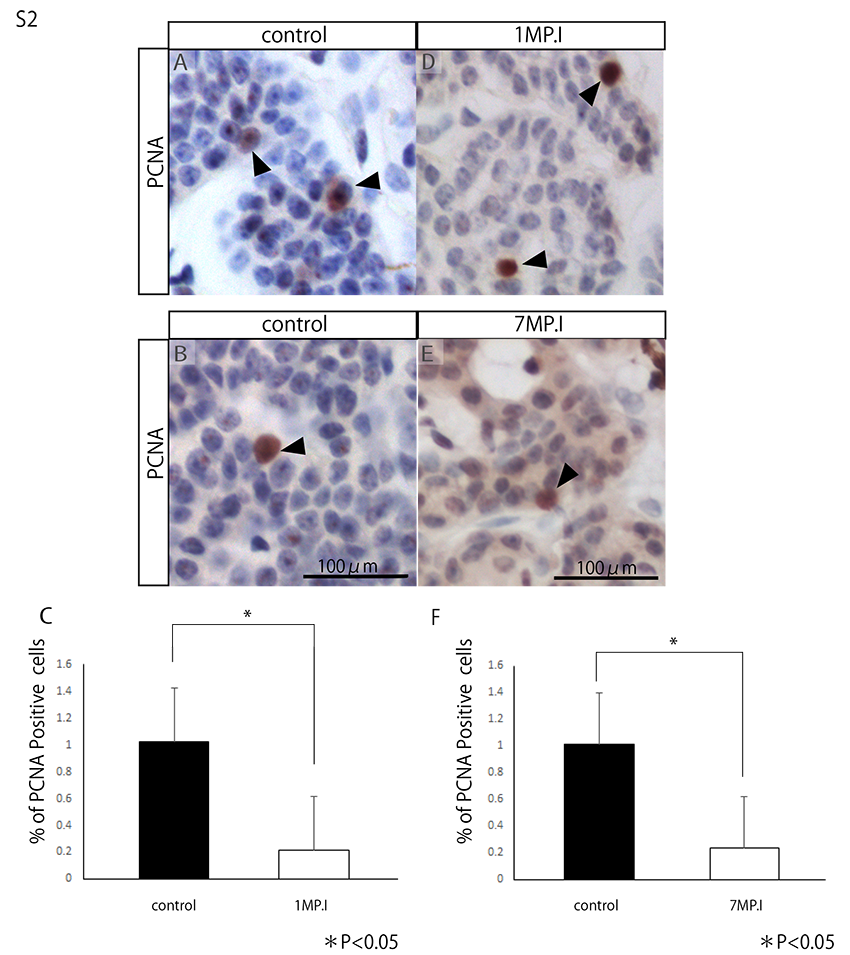

Supplement: S2 Fig — (A, B) PCNA immunostaining of the parathyroid gland tissues in control mice (A) and in one months after administration of tamoxifen (1MP.I) mice (B). Brown-colored cells are PCNA-positive cells, and black arrowheads indicate PCNA-positive parathyroid cells (Scale bar = 100 μm). (C) Control and 1MP.I mice tissues had PCNA-positive cell ratios of 1.02% (n = 5) and 0.21% (n = 5) (U-test, *P < 0.05). (C, D) PCNA immunostaining in the parathyroid glands of control (C) and 7MP.I mice (D). Black arrowheads indicate PCNA-positive parathyroid cells (scale bar = 100 μm). (E) Control and 7MP.I mice tissues had PCNA-positive cell ratios of 1.01% (black bar, n = 5) and 0.23% (white bar, n = 5), respectively (U-test, *P < 0.05). (TIF) [file pone.0210662.s002.tif]

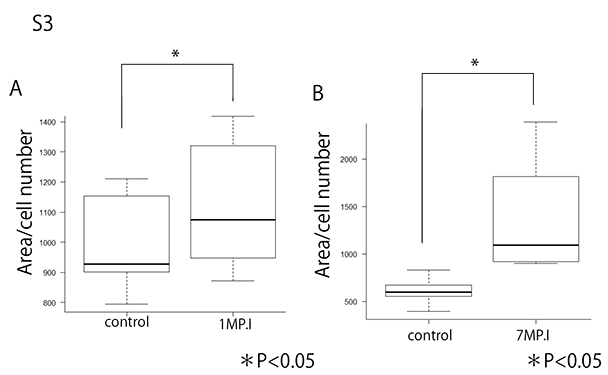

Supplement: S3 Fig — (A) Control and 1MP.I had parathyroid gland area/cell number ratios at 904 (n = 6) and 954 (n = 6), respectively (U-test, *P < 0.05). (B) The area/cell number ratios of parathyroid glands of control and 7MP.I had 929 (n = 7) and 556 (n = 4), respectively (U-test, *P < 0.05). (TIF) [file pone.0210662.s003.tif]

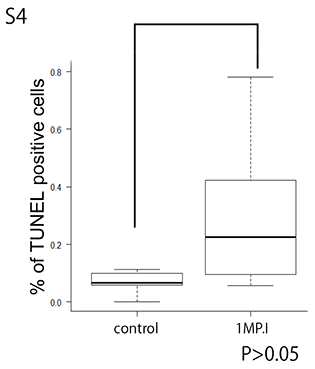

Supplement: S4 Fig — Results were varied, but we found no statistically significant differences between control and 1MP.I mice. (TIF) [file pone.0210662.s004.tif]
